# The airway neuro-immune axis as a therapeutic target in allergic airway diseases

**DOI:** 10.1186/s12931-024-02702-8

**Published:** 2024-02-08

**Authors:** Wanhua Wu, Jianing Li, Su Chen, Suidong Ouyang

**Affiliations:** 1 Guangdong Provincial Key Laboratory of Medical Molecular Diagnostics, The First Dongguan Affiliated Hospital, College of Medical Technology, Guangdong Medical University, Dongguan, 523808 China; 2 Liaobu Hospital of Dongguan City, Dongguan, 523430 China

**Keywords:** Airway neuro-immune axis, Therapeutic target, Asthma, Allergic rhinitis

## Abstract

Recent evidence has increasingly underscored the importance of the neuro-immune axis in mediating allergic airway diseases, such as allergic asthma and allergic rhinitis. The intimate spatial relationship between neurons and immune cells suggests that their interactions play a pivotal role in regulating allergic airway inflammation. Upon direct activation by allergens, neurons and immune cells engage in interactions, during which neurotransmitters and neuropeptides released by neurons modulate immune cell activity. Meanwhile, immune cells release inflammatory mediators such as histamine and cytokines, stimulating neurons and amplifying neuropeptide production, thereby exacerbating allergic inflammation. The dynamic interplay between the nervous and immune systems suggests that targeting the neuro-immune axis in the airway could represent a novel approach to treating allergic airway diseases. This review summarized recent evidence on the nervous system’s regulatory mechanisms in immune responses and identified potential therapeutic targets along the peripheral nerve-immune axis for allergic asthma and allergic rhinitis. The findings will provide novel perspectives on the management of allergic airway diseases in the future.

## Introduction

Allergic airway diseases, such as allergic asthma and allergic rhinitis, are characterized by the prevalence of type 2 helper T (Th2) cells and increased eosinophil infiltration in the airway. While allergic asthma manifests as bronchospasm and airway obstruction, allergic rhinitis presents symptoms such as nasal congestion, rhinorrhea, itching, and sneezing [1, 2]. The immunopathogenic mechanisms underlying allergic airway diseases have been previously elucidated [3, 4]. However, there is emerging evidence indicating the involvement of neurogenic mechanisms in the pathogenesis of allergic airway diseases. Dixon’s research has indicated that the onset of bronchospasm in individuals with asthma is triggered by the activation of neurons in the nasal mucosa, reinforcing the idea that neural mechanisms contribute to the onset of asthma [5]. Moreover, an increase in peptidergic nerves and neuropeptides has been observed in cases of allergic rhinitis [6, 7]. Pharmaceutical compounds, including alpha- and beta-adrenergic agonists, can elicit symptoms of allergic rhinitis. The surgical severing of specific nasal nerves has been demonstrated to alleviate symptoms associated with allergic rhinitis [8, 9]. The findings of these studies suggest that the innervation of peptidergic classes plays a significant role in the neurogenic mechanism of allergic rhinitis.

Recent analysis of the spatial distribution of immune cells and nerve fibers has revealed that the interaction between the immune system and the neurological system regulates allergic airway inflammation, rather than operating independently. Upon activation by cytokines in response to allergens, peripheral neurons convey signals to the central nervous system. This activation induces the release of neuropeptides and neurotransmitters, stimulating neurons through axonal reflexes. Subsequently, neuropeptides and neurotransmitters impact various immune cells, resulting in the generation of inflammatory mediators, including cytokines, lipid mediators, and histamines. Inflammatory mediators, in turn, enhance neuronal excitability, reducing the threshold for neuronal activation in response to stimulation. This establishes a positive feedback loop, activating neurons even in response to subthreshold or non-noxious stimuli, intensifying neuronal activity at the inflammation site [10].

This review summarizes recent findings elucidating the intricate mechanisms by which the nervous system modulates immune responses and identifies potential therapeutic targets for allergic asthma and allergic rhinitis through the airway neuro-immune axis. The insights presented herein provide a novel perspective on the prospective management of allergic airway diseases.

## The distribution and expression of peripheral neurons and immune cells in the airway contribute to the intricate crosstalk between these two systems

The intricate crosstalk between peripheral neurons and immune cells in the airway is closely linked to their distribution and expression patterns. The peripheral nervous system, a crucial component of the broader nervous system, consists of the autonomic and somatic nervous systems. Traditionally, the autonomic nervous system comprises sympathetic and parasympathetic nerves, with the vagus nerve holding a pivotal role in parasympathetic regulation, while the enteric nervous system forms part of the autonomic network [11]. Within the respiratory tract, these nerves play a pivotal role, including sympathetic, parasympathetic, and sensory nerves, with primary distribution in the nasal mucosa. They are instrumental in regulating vasomotor activity, blood flow within the nasal cavity, and the transmission of sensory signals from the nose [12]. In both human and murine lungs, the innervation profile exhibits a dense distribution of parasympathetic nerves, with sympathetic nerves being relatively sparse, constituting approximately 20% of the overall lung innervation [13]. These nerves intricately traverse the trachea, bronchi, blood vessels, and provide support to submucosal glands [14, 15].

In a non-inflammatory state, the airway epithelium and smooth muscle layer harbor a limited number of dendritic cells (DCs) and nerve fibers. Most T cells and DCs are located beneath the smooth muscle, with about 10% of DCs and 4% of T cells forming clusters with sensory nerve fibers. Mast cells, essential components of the immune response, are strategically positioned around microvessels, visceral tissue mucosa, submucosa, and smooth muscle, establishing robust connections with nerves through the expression of cell adhesion molecules or the release of neurotrophic factors and cytokines [16–18]. Furthermore, the presence of neuronal vascular cell adhesion protein 1 and intercellular adhesion molecule 1 suggests that eosinophils may also be attracted to parasympathetic nerves. This extensive distribution profile underscores the potential for intricate interactions between neurons and immune cells in the airway microenvironment [19]. The interplay between their spatial disposition and functional roles provides a foundation for comprehending the nuanced crosstalk within the peripheral neurons-immune cells axis in the airway.

## Peripheral neurons orchestrate airway immune responses during inflammation

The intricate interaction between neurons and immune cells in the airways is orchestrated by primary mediators, encompassing inflammatory mediators, neurotrophins, and neuropeptides. Previous research has predominantly focused on the role of inflammatory mediators in facilitating these interactions. Upon allergen stimulation, epithelial cells release alarmins, such as interleukin (IL)-25, IL-33, and thymic stromal lymphopoietin, directly impacting type 2 innate lymphoid cells (ILC2s). This cascade induces the production of IL-4 and IL-5, activating eosinophils, promoting mast cell proliferation, and stimulating goblet cells, ultimately resulting in airway hyperresponsiveness and increased mucus production. Additionally, IL-4 facilitates DCs in allergen uptake, activating Th2 cells and prompting B lymphocytes to produce allergen-specific IgE antibodies [20, 21].

Cross-linking high-affinity IgE receptors on mast cells and basophils triggers the release of inflammatory mediators, including histamine and prostaglandins. These mediators not only regulate neuronal sensitization by acting on specific membrane receptors but also modulate sodium currents, increasing neuronal excitability. Elevated excitability reduces the stimulation threshold, enabling neurons to be activated by subthreshold or non-injurious stimuli, consequently leading to the release of neuropeptides [22]. Furthermore, small-molecule mediators from other immune cells not only exert pro-inflammatory effects but also directly activate peripheral neurons by binding to receptors expressed on their surface [23, 24].

Studies have highlighted the pivotal role of neurotrophins, including nerve growth factor (NGF), brain-derived neurotrophic factor (BDNF), neurotrophic factor 3 (NT-3), and neurotrophic factor 4/5 (NT-4/5), in mediating interactions between peripheral neurons and immune cells. While higher levels of BDNF have been observed in cases of allergic rhinitis and allergic asthma, further investigations are ongoing to elucidate its impact on nerve fibers. Conversely, NGF has been previously shown to stimulate the formation of nerve fibers and facilitate the infiltration of various immune cells, such as eosinophils and lymphocytes. These immune cells, in turn, trigger Th2 cell-mediated allergic airway inflammation [25, 26].

Neuropeptides play a pivotal role in facilitating communication between peripheral neurons and immune cells. Protease allergens, such as those from house dust mites, directly stimulate TRPV1 + sensory neurons, eliciting the release of neuropeptide SP. This neuropeptide subsequently stimulates DCs and mast cells via Mas-related G-protein coupled receptor members A1 and Mrgprb2, respectively, leading to the generation of pro-inflammatory mediators such as histamine and cytokines. This intricate process triggers additional neuropeptide release from neurons [27, 28]. Consequently, the interaction between neurotransmitters or neuropeptides released by neurons and immune cells in regulating allergic airway inflammation is emerging as a prominent and intriguing area of research.

## Sympathetic nerve-ILC2 crosstalk modulates Th2 responses in allergic asthma

Sympathetic nerves play a crucial role in regulating immune responses by releasing the neurotransmitter norepinephrine (NE), which binds to the β2 adrenergic receptor (β2AR). This interaction acts on airway smooth muscle cells to induce bronchodilation. Consequently, β2 adrenergic receptor agonists are commonly employed as bronchodilators in combination with glucocorticoids to alleviate asthma symptoms and reduce airway inflammation [29, 30]. Recent 3D studies of innervation and immunological responses in lung tissue have unveiled that sympathetic innervation inhibits immune responses mediated by LPS or IL-33 in approximately 20% of total axons in the lung. Further investigations have illuminated that sympathetic nerves mediate the suppression of immune responses through signals binding to β2AR [11]. Parallel studies have identified that β2AR expression on mouse ILC2s co-localizes with adrenergic neurons in the intestine, and the absence of β2AR may lead to excessive activation of ILC2s in lung tissue, resulting in the production of type 2 cytokines and inducing lung inflammation. Conversely, the administration of β2AR agonists resulted in a reduction in inflammation [31]. These findings suggest that norepinephrine released from sympathetic nerves, combined with β2AR expressed on the surface of ILC2s, inhibits immune responses by negatively regulating signaling pathways.

## Parasympathetic nerves-receptors crosstalk regulates type 2 inflammation in allergic asthma

In the pathophysiology of asthma, acetylcholine (ACh) serves as a neurogenic neurotransmitter released by parasympathetic postganglionic neurons. ACh not only induces smooth muscle contraction and mucus secretion but also fosters airway inflammation, contributing to airway remodeling [32]. Within the airway, various types of acetylcholine receptors, including nicotinic and muscarinic receptors, bind to ACh and are expressed on different cells, such as epithelial cells, macrophages, and T cells [33]. Studies have indicated that M3-type muscarinic receptors exacerbate asthma symptoms, primarily located in the airway smooth muscle and submucosal glands, where they regulate smooth muscle contraction and mucus secretion [34]. In contrast, M2 receptors typically inhibit the secretion of ACh, but under stimuli like allergens and viral infections, dysfunctional M2 receptors may increase ACh release, leading to bronchoconstriction and mucus secretion [35]. However, because the ACh released from the vagus nerve binds to different receptors than the parasympathetic-derived acetylcholine, the effects are opposite. When the vagus nerve is stimulated, it suppresses the inflammatory response [36]. After the vagus nerve is deactivated, the pro-inflammatory cytokines increase, exacerbating lung inflammation. These effects are induced by acetylcholine through the α7 nicotinic acetylcholine receptor (α7nAChR) [37]. Furthermore, studies suggest that α7 nicotinic receptor agonists may directly inhibit ILC2s, thereby reducing type 2 inflammatory responses [38]. Therefore, ACh constricts airways via M3 muscarinic receptors while mitigating type 2 inflammation in asthma through nicotinic receptors.

Parasympathetic nerves also release vasoactive intestinal peptide (VIP) in addition to ACh [39]. VIP plays multiple biological roles in mediating immune function by interacting with specific receptors expressed on various immune cells including chemoattractant receptor-homologous molecule expressed on Th2 cells (CRTH2), vasoactive intestinal peptide receptor type 1 (VPAC-1), vasoactive intestinal peptide receptor type 2 (VPAC-2), and pituitary adenylate cyclase activating polypeptide receptors (PAC1) [40, 41]. VIP acts as an effective anti-inflammatory agent to dilate the bronchial tubes [42]. Mice lacking the VIP gene showed airway inflammation and hyperresponsiveness. Asthma symptoms were relieved after intraperitoneal injection of VIP, indicating that VIP plays an anti-inflammatory role in the development of asthma [43, 44]. Conversely, VIP binding to the VPAC2 receptor upregulates IL-5 and IL-13, promotes the accumulation of eosinophils, and induces the conversion of the Th2 phenotype, altering the immune response to allergy and inflammation [45, 46]. Therefore, immune responses mediated by VIP also vary depending on the interacting receptors.

## Sensory nerves and neuroendocrine cells contribute to the pathophysiology of allergic asthma

Upon exposure to allergens, neuropeptides such as substance P (SP), neurokinin A (NKA), and calcitonin gene-related peptide (CGRP) are released from sensory nerves, significantly contributing to the pathophysiology of asthma. Elevated levels of SP are observed in cases of allergic asthma, and its activity is mediated by key receptors, including neurokinin-1 receptors (NK1R) and NK2R. Apart from mediating bronchoconstriction, vasodilation, and increased mucus secretion, SP activates various immune cells, including dendritic cells, macrophages, eosinophils, neutrophils, monocytes, and lymphocytes [28, 47–49]. SP induces inflammation in neutrophils by mediating NK1 receptor-induced synthesis of chemokines such as CCL4 and CXCL8 [50], consistent with findings showing a positive correlation between SP expression and asthma severity [51]. Additionally, SP interacting with the Mrgprb2 receptor/MrgprX2 (human) on mast cells leads to the release of pro-inflammatory cytokines, chemokines, and the recruitment of immune cells like neutrophils, monocytes, and macrophages [27]. NKA, also known as tachykinin, collaborates with SP to promote bronchoconstriction and enhance the antigen presentation function of DCs when combined with the NK2R signal, supporting the immune response [52]. So far, SP and NKA have played roles in promoting inflammation and exacerbating asthma.

**Fig. 1:**
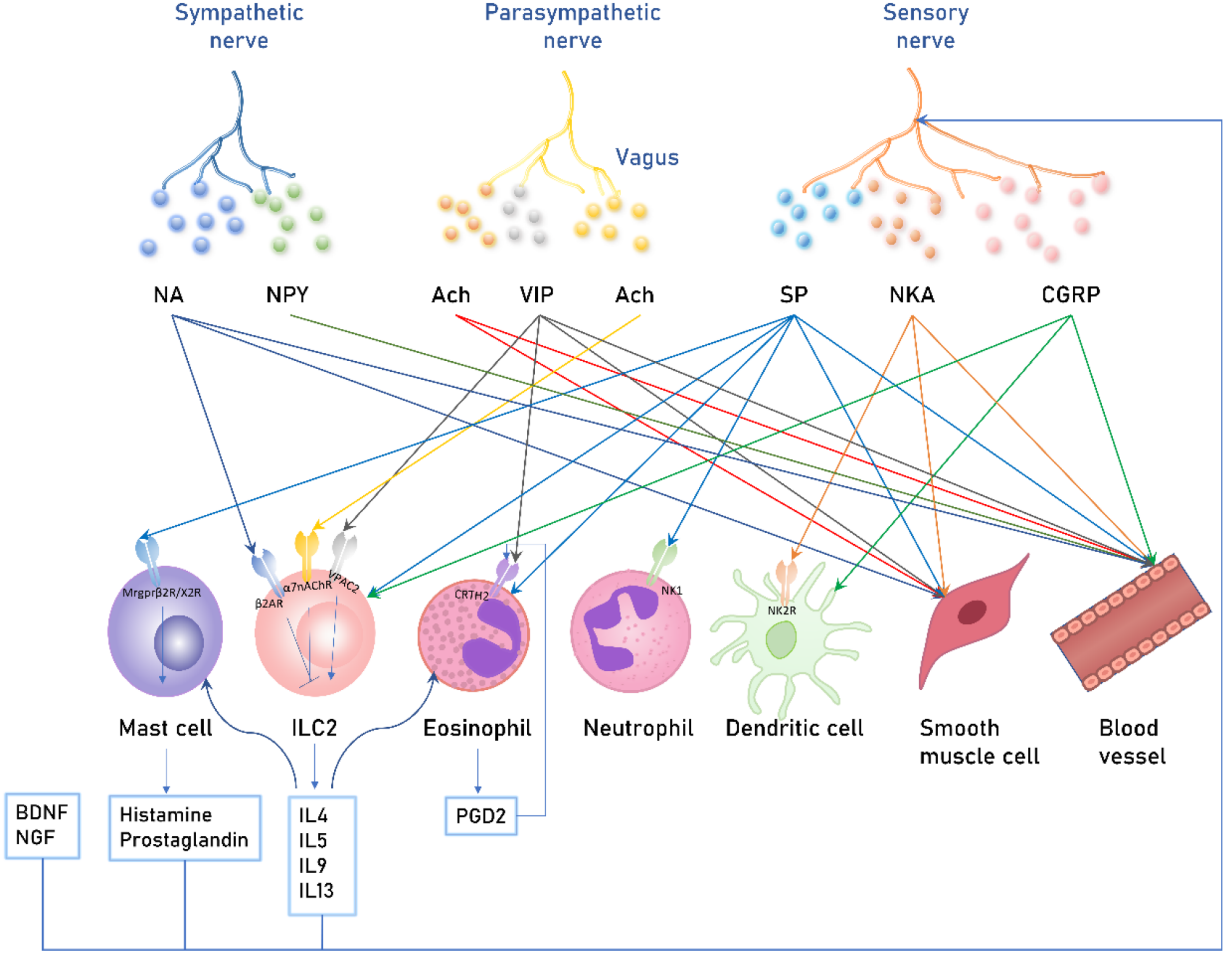
Schematic diagram illustrating the interaction between airway nerve and allergic airway cells

CGRP is synthesized by neuroendocrine cells and stored in sensory nerve terminals. CGRP directly interacts with ILC2s to release IL-5 and IL-13, leading to the recruitment of eosinophils and triggering the Th2 response [53]. Furthermore, CGRP regulates Th9 responses, and stimulates the production of GATA3 and IL-9, amplifying airway inflammation [42]. However, studies demonstrate that CGRP has anti-inflammatory properties affecting the antigen presentation of DCs, inhibiting DC maturation in mouse lungs, subsequently reducing the activation and proliferation of antigen-specific T cells, and increasing the number of regulatory T cells [54]. Research on the multimodal regulation of DC function by nociceptors in specialized sensory nerve endings suggests that CGRP enhances the sentinel function of antigen capture and recognition by DCs in the absence of immune stimulation. In contrast, with simultaneous itching or pain and pathogen presence, activated sensory nerves release the chemokine CCL2, attracting DCs to interact and upregulating the expression of IL-12 p40 and IL-6, shared by IL-12 and IL-23, thereby increasing DC cytokine effects [55]. Therefore, further research on CGRP’s function is necessary (Fig. 1; Table 1).

## Sympathetic nerves influence airway immunity in allergic rhinitis

In addition to norepinephrine, secreted by sympathetic nerves in the nasal mucosa and acting as a potent vasoconstrictor, sympathetic nerves also release neuropeptide Y (NPY). NPY-secreting nerve fibers are predominantly located in arteries and veins, with a smaller number in the epithelium and glands. This distribution suggests that NPY plays a crucial role in regulating blood flow [56]. For example, in the human nasal mucosa, NPY may constrict arteries and regulate vasomotion [57]. Additionally, NPY enhances the vasoconstrictive effects of NE [58]. While sympathetic nerves predominantly maintain vascular tone under normal circumstances, NPY counters the proinflammatory and vasodilatory effects of neurotransmitters and neuropeptides released from parasympathetic and sensory nerves in allergic conditions [59, 60].

## Parasympathetic nerves recruit eosinophils to regulate airway immunity in allergic rhinitis

Parasympathetic fibers intricately innervate the blood vessels and eccrine glands within the nasal mucosa, with a predominant presence in the glands [61]. A comprehensive examination of the neural atlas of the nasal mucosa reveals a significant increase in nerve fibers containing neuropeptides, particularly VIPergic fibers. This emphasizes the significance of VIP innervation in allergic rhinitis [62]. Previous research has elucidated that VIP exerts its biological functions through two receptors, VPAC-1 and VPAC-2 [63]. Recent studies underscore a novel neuroimmune axis, the VIP-CRTH2 axis, which recruits eosinophils. In patients with allergic rhinitis (AR), nasal cavity stimulation leads to a significant increase in neurocrine VIP content, and the secreted VIP recruits eosinophils through the CRTH2 receptor [64]. Furthermore, eosinophils secrete prostaglandin D2 (PGD2), which competes with VIP for CRTH2 receptor signaling, contributing to eosinophil attraction. Leukocytes isolated from nasal secretions of AR patients reveal that CRTH2 is upregulated specifically during the late phase of the response, indicating a crucial role for CRTH2 in the significant recruitment of eosinophils to the inflamed allergic nose [64]. VIP may also modulate the release of acetylcholine, promote vasodilation, and enhance the glandular secretory response, potentially leading to nasal congestion [65].

## Sensory nerves and neuroendocrine cells influence the pathophysiology of allergic rhinitis

Neuropeptides, including SP, NKA, and CGRP, play a significant role in the regulation of allergic rhinitis. Studies have demonstrated a notable increase in the expression of neuropeptides such as CGPG and SP in the nasal cavity of individuals with allergic rhinitis (AR) compared to control participants [66]. SP and NKA act as chemoattractants and activators of various immune cells in AR, leading to cell infiltration and aggravation of inflammation. Additionally, they induce vasodilation in the nasal mucosa, plasma extravasation, and glandular secretion [66, 67]. The application of SP to the nasal mucosa in humans results in elevated mRNA expression of pro-inflammatory cytokines IL-1, IL-3, IL-5, IL-6, TNFα, and IFN-γ. This suggestes that SP may regulate allergic responses by increasing the production of specific regulatory cytokines [68].

In allergic rhinitis, the binding sites of CGRP are mainly located in small arteries, consistent with CGRP serving as a sustained and enduring arterial dilator. Consequently, CGRP may induce prolonged dilation of arterial vessels, leading to increased blood flow in the nasal cavity and subsequently leakage of albumin and plasma water into the nasal cavity [69] (Fig. 1; Table 1).

**Table 1:** Summarizing the impact of airway nerves on targeted cells in allergic airway diseases

Airway nerves	Targeted cells	The impact on allergy airway diseases	Refs
Sympathetic nerves		**Allergic asthma**	
	Airway smooth muscle cells	Bronchodilation	[29, 30]
	ILC2	Reduce type 2 cytokine and attenuate lung inflammation	[31]
		**Allergic rhinitis**	
	Vascular smooth muscle cell	Vasoconstriction	[57]
Parasympathetic nerves		**Allergic asthma**	
	Airway smooth muscle cells	Bronchoconstriction/bronchodilation	[32, 42]
	Epithelial cells, Macrophages, Eosinophils	Mucus secretion Inhibit airway hyperresponsiveness and airway inflammation Upregulate IL-5 and IL-13, promotes the accumulation of eosinophils	[34, 43–46]
	ILC2s	Reduce pro-inflammatory cytokines production	[36–38]
		**Allergic rhinitis**	
	Eosinophils, Lymphocytes	Recruit eosinophils	[64]
	Vascular smooth muscle cell	Vasodilation	[65]
Sensory nerves and neuroendocrine cells		**Allergic asthma**	
	Smooth muscle cell	Bronchoconstriction, vasodilation, and increased mucus secretion	[47]
		**Allergic rhinitis**	
		Vasodilation in the nasal mucosa, plasma extravasation, and glandular secretion	[66, 67, 69]
		**Allergic asthma**	
	Mast cell, ILC2s, Eosinophils, Monocytes Dendritic cells, Macrophages,	Release pro-inflammatory cytokines, chemokines, and recruit immune cells Affect the antigen presentation function of DCs	[27, 28, 48–53]
		**Allergic rhinitis**	
	Neutrophils	Release pro-inflammatory cytokines, chemokines, and recruit immune cells	[68]

## The clinical application of the airway neuro-immune axis as a therapeutic target in allergic airway diseases

A potential starting point for treating allergic airway diseases lies in critical aspects of clinical therapy. Emerging research on the interaction between the nervous system and the immune system provides novel insights and approaches for allergic respiratory diseases. Generally, drugs for treating allergic airway diseases can be categorized into three main groups:

### Drugs targeting inflammatory mediators and receptors released by immune cells

In the management of allergic airway diseases, medications targeting inflammatory mediators released by immune cells include TSLP mAb, IL-5 mAb, IL-13 mAb, and IL-33 mAb. Drugs that target pro-inflammatory mediator receptors encompass histamine Type 1/2 receptor antagonists and IL-4 receptor alpha (IL4-Ra) mAb. Recently, the development of drugs targeting chemokine receptors acting on CCR3 has shown promise. Studies have introduced a novel peptide nanoparticle CCR3 inhibitor effectively preventing eosinophil recruitment to the lungs and airways, along with mitigating airway hyperresponsiveness, thereby reducing inflammation [70].

### Drugs targeting ion channels that activate neurons

Drugs targeting ion channels activating neurons are also under investigation. The Transient Receptor Potential Cation Channel Subfamily V Member 1 (TRPV1) is a crucial ion channel receptor in sensory neurons, and its overexpression induced by eosinophils can be mitigated by capsaicin binding to TRPV1 [71]. Conversely, treatment with a TRPV1 antagonist reduces airway hyperresponsiveness and inflammation [63].

### Drugs targeting neurotransmitters and neuropeptides released by neurons and their receptors

Drugs that target neurotransmitters and neuropeptides released from neurons and their receptors have broad applications in influencing neuroimmune interactions. Clinically, the standard treatment drugs for asthma include short-acting or long-acting β2-receptor agonists (such as albuterol and formoterol) because β2 receptor agonists have been traditionally recognized as bronchodilators. Recent studies have revealed that the signaling of norepinephrine released from pulmonary sympathetic nerves to β2 receptors can not only dilate airways but also reduce lung inflammation through favorable immunomodulatory effects [13, 72]. The mechanism of action may be that the signaling of β2-adrenergic receptors inhibits the function of ILC2s [31]. In addition, medications that target acetylcholine receptors, such as inhaled selective M3 receptor antagonists used as bronchodilators, are currently employed in the treatment of asthma and COPD [73, 74]. In cases of allergic rhinitis and allergic asthma, where eosinophil inflammation is predominant, the use of anti-CRTH2 antibodies is beneficial. This is because eosinophils express CRTH2 at high levels, but not VPAC1 or VPAC2, leading to a significant decrease in VIP binding [40].

Recently, neuropeptides have been discovered as a new therapeutic target. The exogenous administration of α-CGRP reduces the level of eosinophils and increases the production of regulatory T cells, suggesting that CGRP may serve as an anti-inflammatory mediator and a new target for therapy [54, 75]. Because VIP serves a variety of biological purposes, VIP analogs and/or antagonists may offer improved therapeutic alternatives for treating allergic diseases [76, 77]. The role of SP receptor antagonists and NK1R antagonists in human clinical research is unclear [78]. However, studies have demonstrated that NK2 receptor antagonists (such as tachykinin) partially inhibit NKA-induced bronchoconstriction in asthmatic patients. As a result, tachykinin receptor antagonists may be used to treat asthma [35]. All of these medications influence neurotransmitters and neuropeptides, either directly or indirectly, inhibiting the inflammatory response and relieving allergy symptoms.

Moreover, alternative treatments such as psychotherapy and neurorehabilitation may contribute to managing allergic airway diseases. Studies have demonstrated a direct relationship between asthma onset/severity and the patient’s mood and stress. Negative emotions’ impact on the brain can increase systemic inflammation and pro-inflammatory cytokines [79]. Psychotherapy or neurorehabilitation methods, such as meditation, exercise, and social interaction, can reduce inflammation by modulating neural circuit activity and the function of brain centers involved in asthma [80].

## Conclusions

As our scientific exploration into allergic airway diseases advances, we transcend the conventional view of attributing them solely to the autonomous functions of the immune or nervous system. The revelation of the airway neuro-immune axis brings forth novel perspectives on allergic airway diseases, emphasizing the crucial role of the nervous system in regulating immune responses, thus influencing drug treatments. Despite significant progress in immune-targeted therapy for allergic airway inflammation, drug resistance poses a substantial challenge. Therefore, future research aims to delve deeper into this field, seeking a more profound comprehension of the cellular and molecular mechanisms through which the nervous system regulates immune responses via the airway neuro-immune axis. This exploration holds the potential to unveil more effective therapeutic options for managing allergic airway diseases.

## Data Availability

No datasets were generated or analysed during the current study.

## Abbreviations

ACh: Acetylcholine
α7nAChR: α7 nicotinic acetylcholine receptor
AR: Allergic rhinitis
BDNF: Brain-derived neurotrophic factor
β2AR: β2 adrenergic receptor
CGRP: Calcitonin gene-related peptide
CRTH2: Chemoattractant receptor-homologous molecule expressed on Th2 cells
DCs: Dendritic cells
ILC2s: Type 2 innate lymphoid cells
IL-25: Interleukin (IL)-25
IL4-Ra: IL-4 receptor alpha
NE: Norepinephrine
NGF: Nerve growth factor
NKA: Neurokinin A
NK1R: Neurokinin-1 receptors
NPY: Neuropeptide Y
NT: Nneurotrophic factor
PAC1: Pituitary adenylate cyclase activating polypeptide receptors
PGD2: Prostaglandin D2
SP: Substance P
Th2: Type 2 helper T
TRPV1: Transient Receptor Potential Cation Channel Subfamily V member 1
VIP: Vasoactive intestinal peptide
VPAC-1/2: Vasoactive intestinal peptide receptor type 1/2
